# Supplementary material for: Discovering allatostatin type-C receptor specific agonists
Source: Nat Commun. 2024 May 10;15:3965. doi: 10.1038/s41467-024-48156-w (PMC11087482; doi:10.1038/s41467-024-48156-w)
Supplement: Supplementary file 1 — Supplementary Information [file 41467_2024_48156_MOESM1_ESM.pdf]

Supplementary Information

Supplementary Table 1. Docking scores and average MM/GBSA binding free energy values of hit molecules from ChemDiv's GPCR-Targeted and Peptidomimetic libraries and decoy molecules from GPCR Decoy Database (GDD).

Library	Ligand	Docking Score (kcal/mol)	Average MM/GBSA ΔG Score (kcal/mol)
ChemDiv Peptidomimetics Library	J100-0311	-8.497	-101.368 ± 10.414
ChemDiv GPCR-Targeted Library	V007-0853	-8.401	-100.975 ± 10.605
ChemDiv GPCR-Targeted Library	D074-0013	-8.981	-93.583 ± 9.869
ChemDiv GPCR-Targeted Library	V029-3547	-8.418	-92.805 ± 10.536
ChemDiv GPCR-Targeted Library	D074-0034	-9.090	-91.781 ± 8.465
ChemDiv GPCR-Targeted Library	V023-0518	-8.628	-87.555 ± 9.477
ChemDiv GPCR-Targeted Library	V022-4271	-8.572	-86.581 ± 10.013
ChemDiv GPCR-Targeted Library	C794-1617	-8.766	-74.266 ± 13.032
ChemDiv GPCR-Targeted Library	C300-0328	-8.482	-72.890 ± 8.293

ChemDiv GPCR-Targeted Library	V014-0754	-8.970	-57.130 ± 10.699
GDD	ZINC49679835	-7.556	-88.312 ± 9.826
GDD	ZINC29563797	-6.662	-86.281 ± 8.659
GDD	ZINC27549459	-7.972	-82.035 ± 9.308
GDD	ZINC28377245	-7.527	-81.48 ± 9.578
GDD	ZINC42808729	-7.118	-78.751 ± 10.607
GDD	ZINC49756093	-6.727	-78.674 ± 8.208
GDD	ZINC49694457	-7.766	-77.856 ± 9.441
GDD	ZINC40933977	-6.775	-69.534 ± 15.405
GDD	ZINC27999192	-7.002	-40.901 ±8.111
GDD	ZINC26728131	-6.771	-27.949 ± 24.938

Supplementary Table 2. SMILES codes of hit molecules.

Top 10 Molecules	SMILES
**D074-0034**	Oc(cccc1)c1-c1n[nH]c2c1C(c1cc(OCc3ccccc3)ccc1)N(CC1OCCC1)C2=O
**D074-0013**	Oc(cccc1)c1-c1n[nH]c2c1C(c1cc(Oc3ccccc3)ccc1)N(CC1OCCC1)C2=O
**V014-0754**	CCCN(CC(COCc1ccco1)O)CC(N(CCc1c[nH]c2c1cccc2)Cc1ccc(C(C)(C)C)cc1)=O
**C794-1617**	CCOc(cc1)ccc1NCc1cccn1-c1nnc(N(CC2)CCC2C(NC2CCN(Cc3ccccc3)CC2)=O)s1
**V023-0518**	Cc(cc1)ccc1C(Nc1cccc(C(C(C(NCCc2ncccc2)=O)N2Cc3cccc(Cl)c3)OC2=O)c1)=O
**V022-4271**	Cc1c(CN(C(C(c(cc2)ccc2NC(c2ccccc2)=O)O2)C(NCCc3ncccc3)=O)C2=O)cccc1
**J100-0311**	OC1=Nc(cccc2)c2N(CCNC(CC(C(Nc2c3cccc2)=O)NC3=O)=O)C1=O
**C300-0328**	O=C(CCS(c(cc(c(CC1)c2)N1C(C1CC1)=O)c2Br)(=O)=O)NCCN(CC1)CCN1c1ccccc1
**V029-3547**	CCCN(CC(COCc1ccco1)O)CC(N(CCc1c[nH]c2c1cccc2)Cc1ccc(C(C)(C)C)cc1)=O
**V007-0853**	Cc1ccc(Cn2c(cccc3)c3c(C(CC(NCc3ccncc3)=O)c(cc3)cc(OCc4ccccc4)c3OC)c2)cc1

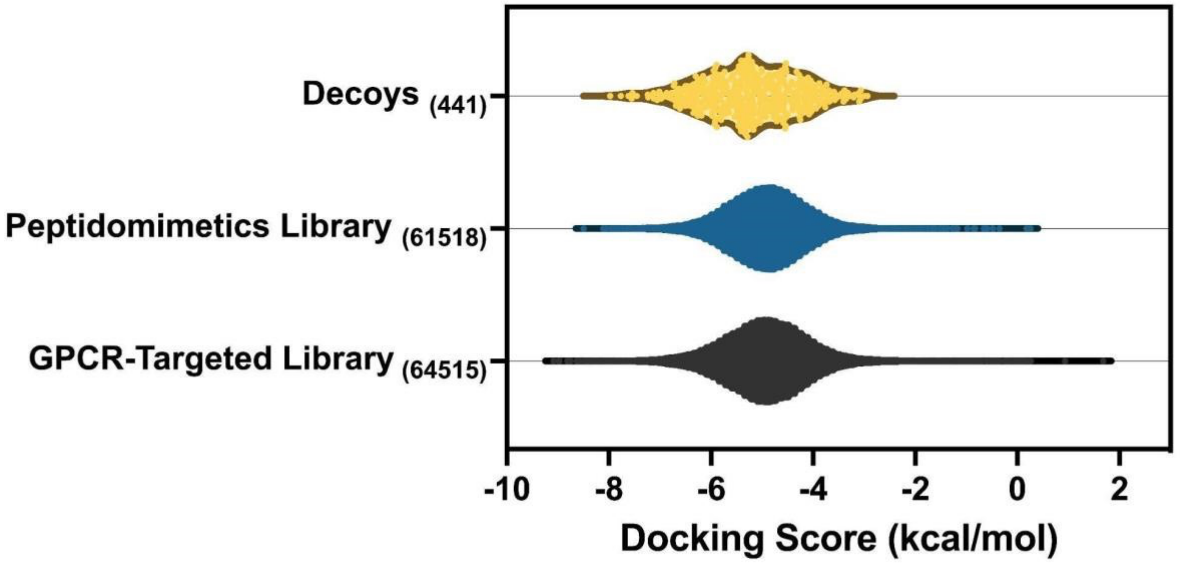

Supplementary Figure 1. Docking scores of compound libraries used in virtual screening. Numbers in parentheses represent the total number of compounds docked in each library. Docking scores are calculated in kcal/mol.

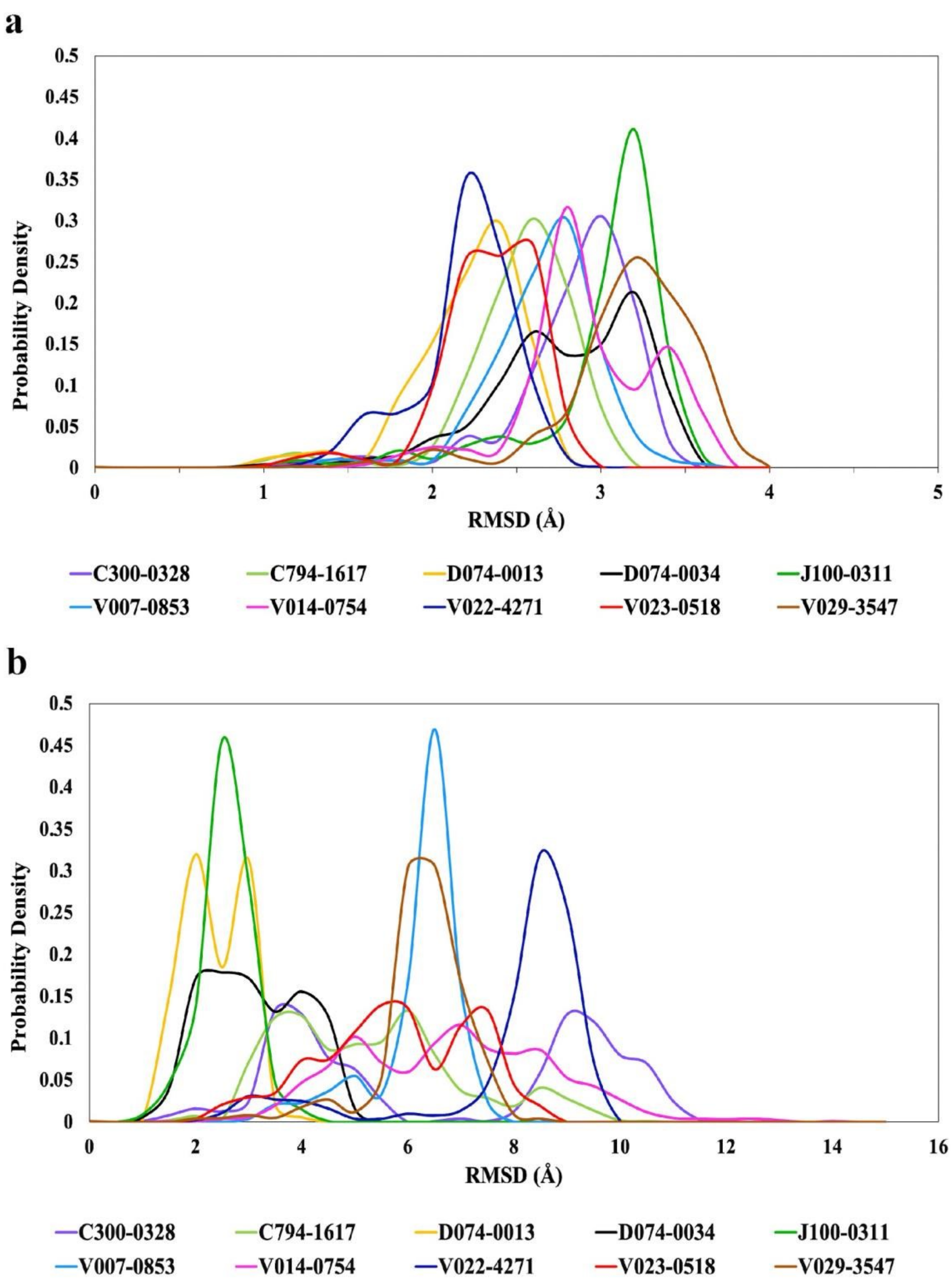

Supplementary Figure 2. System stabilities during MD simulations. a RMSD plot of protein Cα atoms when N-terminus and loops are not included. **b** Ligand-fit-protein (LigFitProt) RMSD plot of systems when N-terminus and loops are not included. LigFitProt represents the RMSD of a

ligand when the protein-ligand complex is first aligned on the protein backbone of the reference and then the RMSD of the ligand heavy atoms is measured.

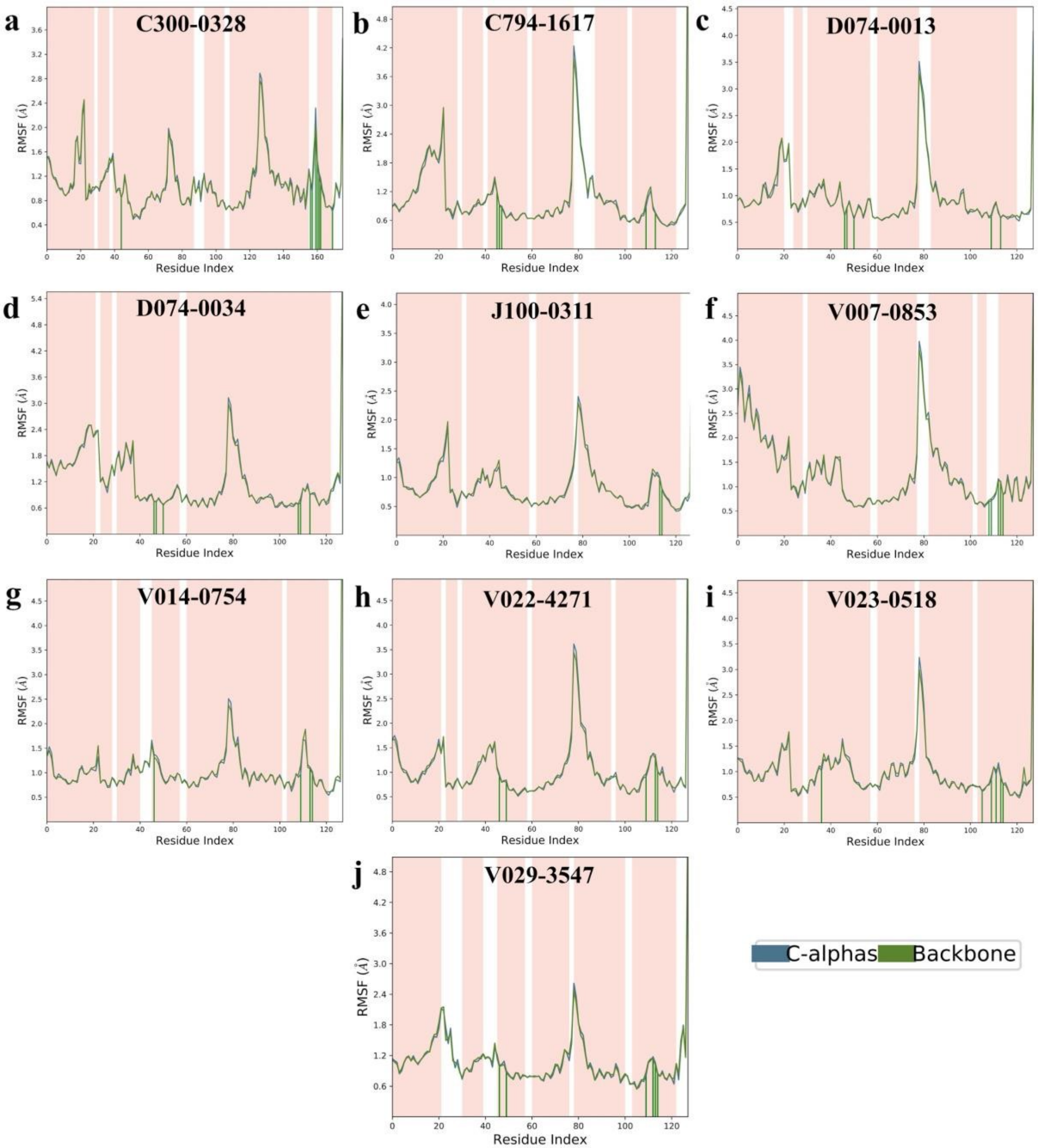

Supplementary Figure 3. The system stability was evaluated considering RMSF changes. (aj) RMSF changes during 100 ns simulation when N-terminus and loops are not included.

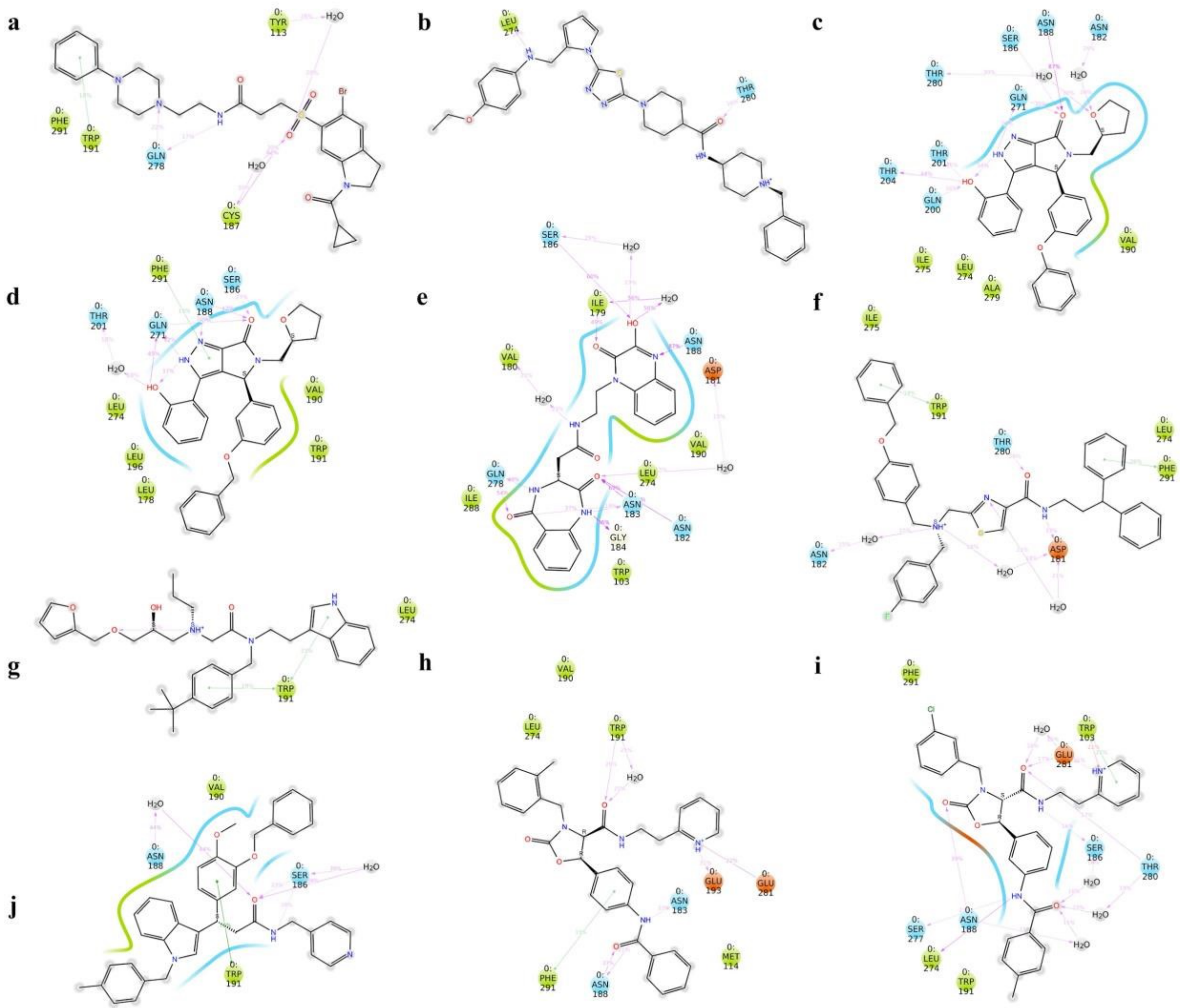

Supplementary Figure 4. 2D representations of the protein−ligand interactions. a C300-0328, **b** C794-1617, **c** D074-0013, **d** D074-0034, **e** J100-0311, **f** V007-0853, **g** V014-1754, **h** V022-4271, **i** V023-0518, **j** V029-3547. The residues of the receptor surrounding the ligands are represented with different colors to show the type of interaction. Lime: hydrophobic interactions, blue: polar contacts, red: charged interactions, dark green: π-π stacking.

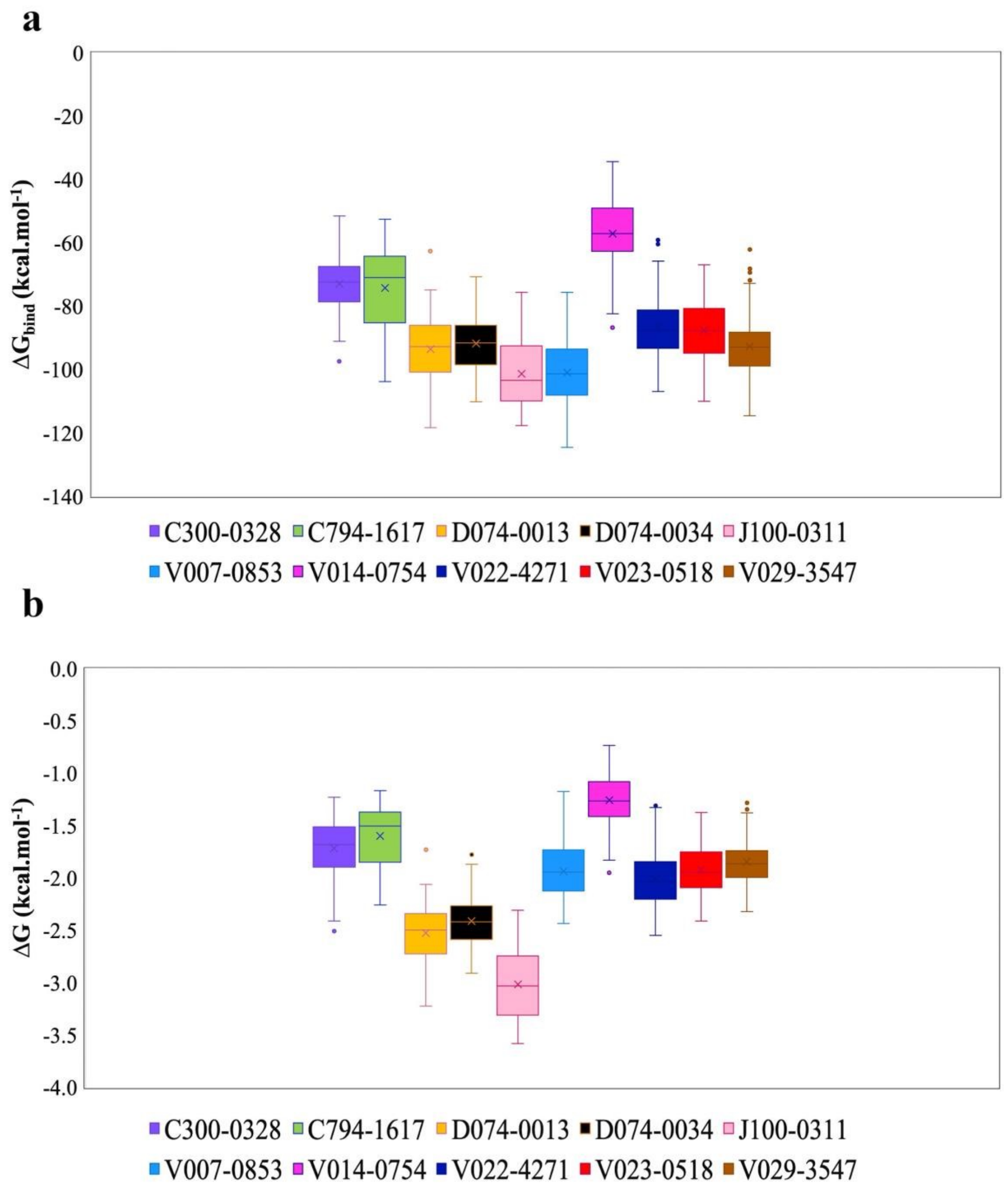

Supplementary Figure 5. Binding free energies of protein-ligand complexes. a ΔG no strain binding free energies of protein-ligand complexes. **b** Ligand efficiency scores of compounds, representing the binding energy of each non-hydrogen ligand atom.

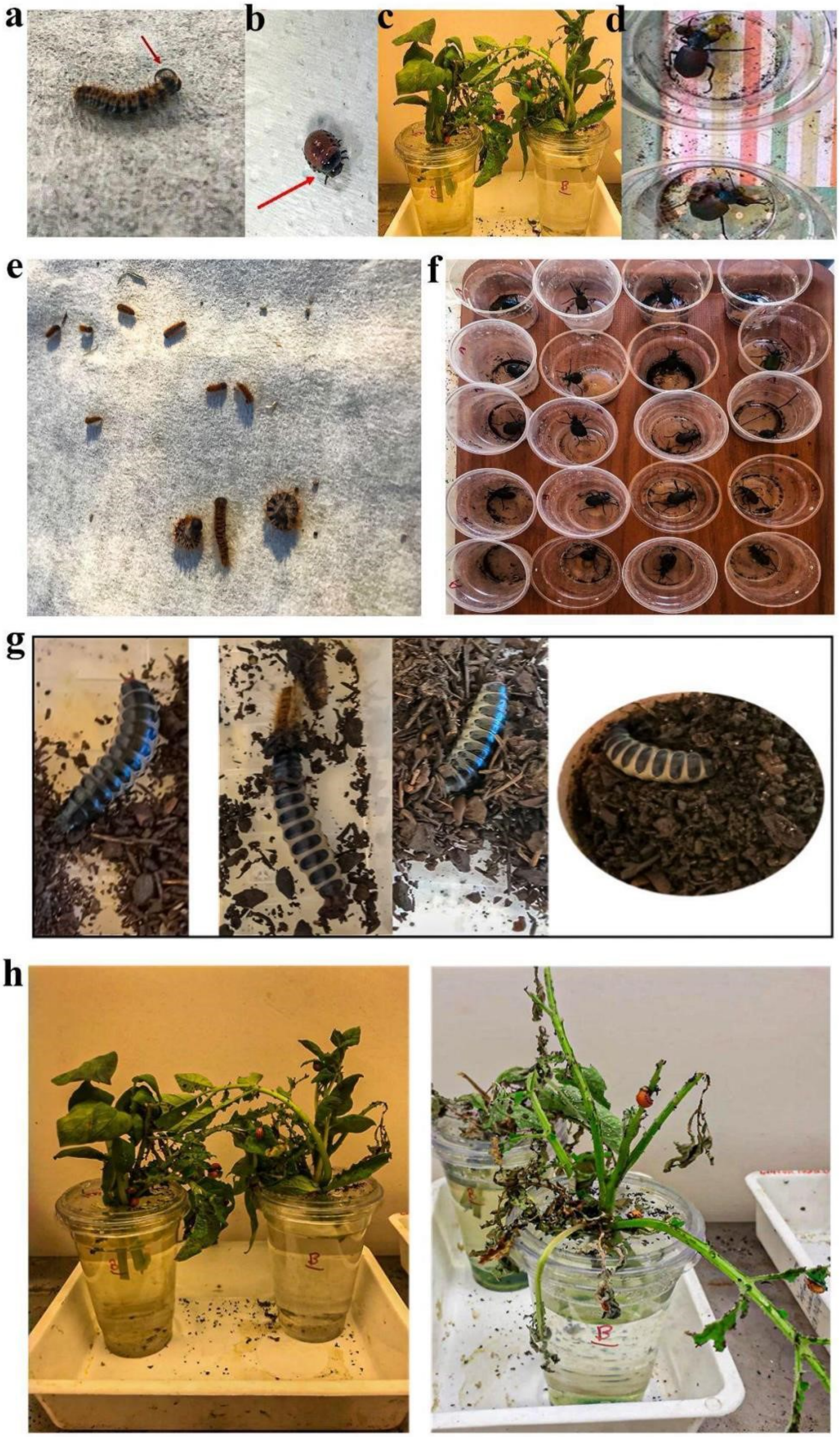
a
b
c
d
e
f
g
h

Supplementary Figure 6. Insects before and after treated with pesticide candidates. a Topical application of molecules to the dorsal surface of the intersection of the potato beetle's headprothorax. **b** Topical application of molecules to the dorsal surface of the intersection of larvae's head-prothorax. **c** Feeding of larvae subjected to application on the dorsal surface of the intersection of the headprothorax on potato plant leaves. **d** Placing *C. sycophanta* adults that have undergone application into cups containing *T. pit* larvae. **e** Outcomes from toxicity tests are delineating the presence of deceased and succeeding (bottom three) *T. pit* larvae. **f** *C. sycophanta* adults were treated with agonists D074-0034, J100-0311, V029-3547, and D074-0013 at a dose of 1000 ppm. **g** Image of *C. sycophanta* larvae 14 days after applying molecules at a dose of 1000 ppm. **h** Left: Potato beetles on potato plant 7 days after applying D074-0034. Right: Potato beetles on potato plant 14 days after applying D074-0034.
