# Supplementary material for: Discovering allatostatin type-C receptor specific agonists
Source: Nat Commun. 2024 May 10;15:3965. doi: 10.1038/s41467-024-48156-w (PMC11087482; doi:10.1038/s41467-024-48156-w)
Supplement: Supplementary file 5 — Source Data [file 41467_2024_48156_MOESM5_ESM.zip › source-data/Source_Data/Source_Data_Supplementary_Data_Tables_1-4.docx]

**Source Data Supplementary Data Table 1.** **Tests of between-subjects effects on *T. pityocampa* larva length.**

| **Tests of Between-Subjects Effects** | | | | | | | | |
| --- | --- | --- | --- | --- | --- | --- | --- | --- |
| Dependent Variable: Larva Length | | | | | | | | |
| Source | Type III Sum of Squares | df | Mean Square | F | Sig. | Partial Eta Squared | Noncent. Parameter | ^Observed Powerb^ |
| Corrected Model | 1238,063^a^ | 5 | 247,613 | 19,926 | ,000 | ,466 | 99,629 | 1,000 |
| Intercept | 7657,616 | 1 | 7657,616 | 616,224 | ,000 | ,844 | 616,224 | 1,000 |
| molecule | 1238,063 | 5 | 247,613 | 19,926 | ,000 | ,466 | 99,629 | 1,000 |
| Error | 1416,641 | 114 | 12,427 |  |  |  |  |  |
| Total | 10312,320 | 120 |  |  |  |  |  |  |
| Corrected Total | 2654,704 | 119 |  |  |  |  |  |  |
| a. R Squared = ,466 (Adjusted R Squared = ,443) | | | | | | | | |
| b. Computed using alpha = ,05 | | | | | | | | |

**Source Data Supplementary Data Table 2.** **One-way ANOVA tests the effects of molecule treatment on *T. pityocampa* larvae to larva length.**

| **Multiple Comparisons** | | | | | | |
| --- | --- | --- | --- | --- | --- | --- |
| Dependent Variable: Larva Length | | | | | | |
| Tukey HSD | | | | | | |
| (I) 14 day-mm | (J) 14 day-mm | Mean Difference (I-J) | Std. Error | Sig. | 95% Confidence Interval | |
|  |  |  |  |  | Lower Bound | Upper Bound |
| AST-C | Control | -9,070* | 1,1147 | ,000 | -12,301 | -5,839 |
|  | D074-0013 | -4,625* | 1,1147 | ,001 | -7,856 | -1,394 |
|  | D074-0034 | -1,225 | 1,1147 | ,881 | -4,456 | 2,006 |
|  | J100-0311 | -1,055 | 1,1147 | ,933 | -4,286 | 2,176 |
|  | V029-3547 | -,365 | 1,1147 | ,999 | -3,596 | 2,866 |
| Control | AST-C | 9,070* | 1,1147 | ,000 | 5,839 | 12,301 |
|  | D074-0013 | 4,445* | 1,1147 | ,002 | 1,214 | 7,676 |
|  | D074-0034 | 7,845* | 1,1147 | ,000 | 4,614 | 11,076 |
|  | J100-0311 | 8,015* | 1,1147 | ,000 | 4,784 | 11,246 |
|  | V029-3547 | 8,705* | 1,1147 | ,000 | 5,474 | 11,936 |
| D074-0013 | AST-C | 4,625* | 1,1147 | ,001 | 1,394 | 7,856 |
|  | Control | -4,445* | 1,1147 | ,002 | -7,676 | -1,214 |
|  | D074-0034 | 3,400* | 1,1147 | ,033 | ,169 | 6,631 |
|  | J100-0311 | 3,570* | 1,1147 | ,021 | ,339 | 6,801 |
|  | V029-3547 | 4,260* | 1,1147 | ,003 | 1,029 | 7,491 |
| D074-0034 | AST-C | 1,225 | 1,1147 | ,881 | -2,006 | 4,456 |
|  | Control | -7,845* | 1,1147 | ,000 | -11,076 | -4,614 |
|  | D074-0013 | -3,400* | 1,1147 | ,033 | -6,631 | -,169 |
|  | J100-0311 | ,170 | 1,1147 | 1,000 | -3,061 | 3,401 |
|  | V029-3547 | ,860 | 1,1147 | ,972 | -2,371 | 4,091 |
| J100-0311 | AST-C | 1,055 | 1,1147 | ,933 | -2,176 | 4,286 |
|  | Control | -8,015* | 1,1147 | ,000 | -11,246 | -4,784 |
|  | D074-0013 | -3,570* | 1,1147 | ,021 | -6,801 | -,339 |
|  | D074-0034 | -,170 | 1,1147 | 1,000 | -3,401 | 3,061 |
|  | V029-3547 | ,690 | 1,1147 | ,989 | -2,541 | 3,921 |
| V029-3547 | AST-C | ,365 | 1,1147 | ,999 | -2,866 | 3,596 |
|  | Control | -8,705* | 1,1147 | ,000 | -11,936 | -5,474 |
|  | D074-0013 | -4,260* | 1,1147 | ,003 | -7,491 | -1,029 |
|  | D074-0034 | -,860 | 1,1147 | ,972 | -4,091 | 2,371 |
|  | J100-0311 | -,690 | 1,1147 | ,989 | -3,921 | 2,541 |
| Based on observed means.  The error term is Mean Square(Error) = 12,427. | | | | | | |
| *. The mean difference is significant at the ,05 level. | | | | | | |

**Source Data Supplementary Data Table 3. Tests of between-subjects effects on *T. pityocampa* head capsule width.**

| **Tests of Between-Subjects Effects** | | | | | | | | |
| --- | --- | --- | --- | --- | --- | --- | --- | --- |
| Dependent Variable: Head Capsule Width | | | | | | | | |
| Source | Type III Sum of Squares | df | Mean Square | F | Sig. | Partial Eta Squared | Noncent. Parameter | Observed Power^b^ |
| Corrected Model | 21.328^a^ | 5 | 4.266 | 13.179 | .000 | .366 | 65.897 | 1.000 |
| Intercept | 292.656 | 1 | 292.656 | 904.240 | .000 | .888 | 904.240 | 1.000 |
| Compound | 21.328 | 5 | 4.266 | 13.179 | .000 | .366 | 65.897 | 1.000 |
| Error | 36.896 | 114 | .324 |  |  |  |  |  |
| Total | 350.880 | 120 |  |  |  |  |  |  |
| Corrected Total | 58.224 | 119 |  |  |  |  |  |  |
| a. R Squared = .366 (Adjusted R Squared = .339) | | | | | | | | |
| b. Computed using alpha = .05 | | | | | | | | |

**Source Data Supplementary Table 4. One-way ANOVA tests the effects of molecule treatment on *T. pityocampa* larvae to head capsule width.**

| **Multiple Comparisons** | | | | | | |
| --- | --- | --- | --- | --- | --- | --- |
| Dependent Variable: head capsule width | | | | | | |
| Tukey HSD | | | | | | |
| (I) 14 day-mm | (J) 14 day-mm | Mean Difference (I-J) | Std. Error | Sig. | 95% Confidence Interval | |
|  |  |  |  |  | Lower Bound | Upper Bound |
| AST-C | Control | -1,105* | ,1799 | ,000 | -1,626 | -,584 |
|  | D074-0013 | -,645* | ,1799 | ,006 | -1,166 | -,124 |
|  | D074-0034 | -,105 | ,1799 | ,992 | -,626 | ,416 |
|  | J100-0311 | -,030 | ,1799 | 1,000 | -,551 | ,491 |
|  | V029-3547 | ,015 | ,1799 | 1,000 | -,506 | ,536 |
| Control | AST-C | 1,105* | ,1799 | ,000 | ,584 | 1,626 |
|  | D074-0013 | ,460 | ,1799 | ,117 | -,061 | ,981 |
|  | D074-0034 | 1,000* | ,1799 | ,000 | ,479 | 1,521 |
|  | J100-0311 | 1,075* | ,1799 | ,000 | ,554 | 1,596 |
|  | V029-3547 | 1,120* | ,1799 | ,000 | ,599 | 1,641 |
| D074-0013 | AST-C | ,645* | ,1799 | ,006 | ,124 | 1,166 |
|  | Control | -,460 | ,1799 | ,117 | -,981 | ,061 |
|  | D074-0034 | ,540* | ,1799 | ,038 | ,019 | 1,061 |
|  | J100-0311 | ,615* | ,1799 | ,011 | ,094 | 1,136 |
|  | V029-3547 | ,660* | ,1799 | ,005 | ,139 | 1,181 |
| D074-0034 | AST-C | ,105 | ,1799 | ,992 | -,416 | ,626 |
|  | Control | -1,000* | ,1799 | ,000 | -1,521 | -,479 |
|  | D074-0013 | -,540* | ,1799 | ,038 | -1,061 | -,019 |
|  | J100-0311 | ,075 | ,1799 | ,998 | -,446 | ,596 |
|  | V029-3547 | ,120 | ,1799 | ,985 | -,401 | ,641 |
| J100-0311 | AST-C | ,030 | ,1799 | 1,000 | -,491 | ,551 |
|  | Control | -1,075* | ,1799 | ,000 | -1,596 | -,554 |
|  | D074-0013 | -,615* | ,1799 | ,011 | -1,136 | -,094 |
|  | D074-0034 | -,075 | ,1799 | ,998 | -,596 | ,446 |
|  | V029-3547 | ,045 | ,1799 | 1,000 | -,476 | ,566 |
| V029-3547 | AST-C | -,015 | ,1799 | 1,000 | -,536 | ,506 |
|  | Control | -1,120* | ,1799 | ,000 | -1,641 | -,599 |
|  | D074-0013 | -,660* | ,1799 | ,005 | -1,181 | -,139 |
|  | D074-0034 | -,120 | ,1799 | ,985 | -,641 | ,401 |
|  | J100-0311 | -,045 | ,1799 | 1,000 | -,566 | ,476 |
| Based on observed means.  The error term is Mean Square(Error) = ,324. | | | | | | |
| *. The mean difference is significant at the ,05 level. | | | | | | |
