# Supplementary material for: Discovering allatostatin type-C receptor specific agonists
Source: Nat Commun. 2024 May 10;15:3965. doi: 10.1038/s41467-024-48156-w (PMC11087482; doi:10.1038/s41467-024-48156-w)
Supplement: Supplementary file 5 — Source Data [file 41467_2024_48156_MOESM5_ESM.zip › source-data/Source_Data/Spectra/FirstSide/V029-3547.PDF]

Sample: 165	Date: 21-Jun-2011	Page 1.
File: Ar39292ac_65	Time: 06:03:37	AMRI code: ALB-H01006214
Vial: A/9	Description: 10009342	Vial label: C1004907AMP0002

(1) ELSD Signal

max. intensity: 1.3E3

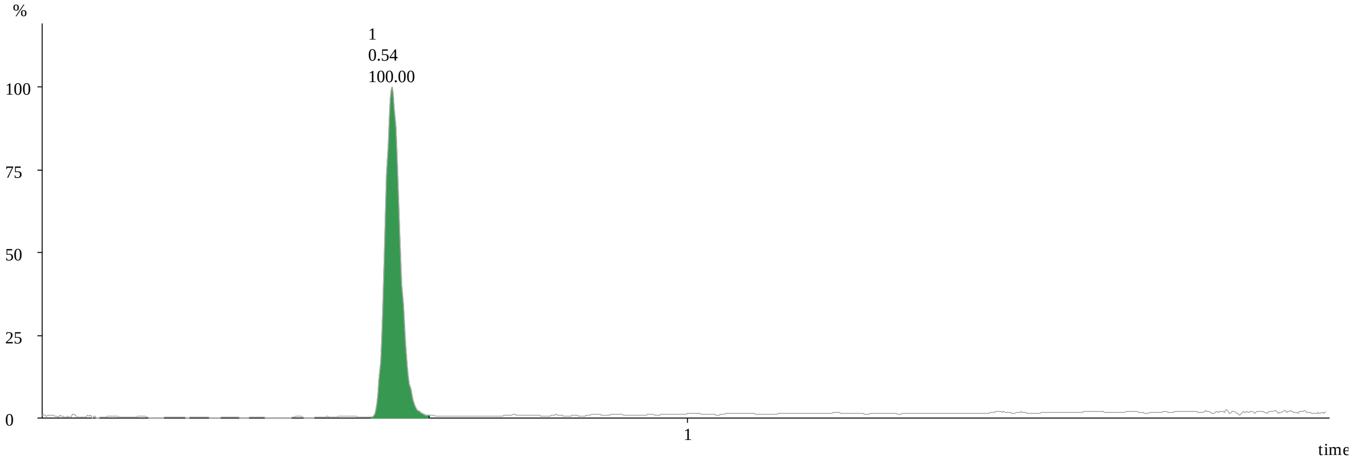

Peak_ID	Peak	Area	Area%	Height	Time	Mass Found
1	0.51 0.60	3.E1	100	1.E3	0.54	595.28

DAD: 220

max. intensity: 2E6

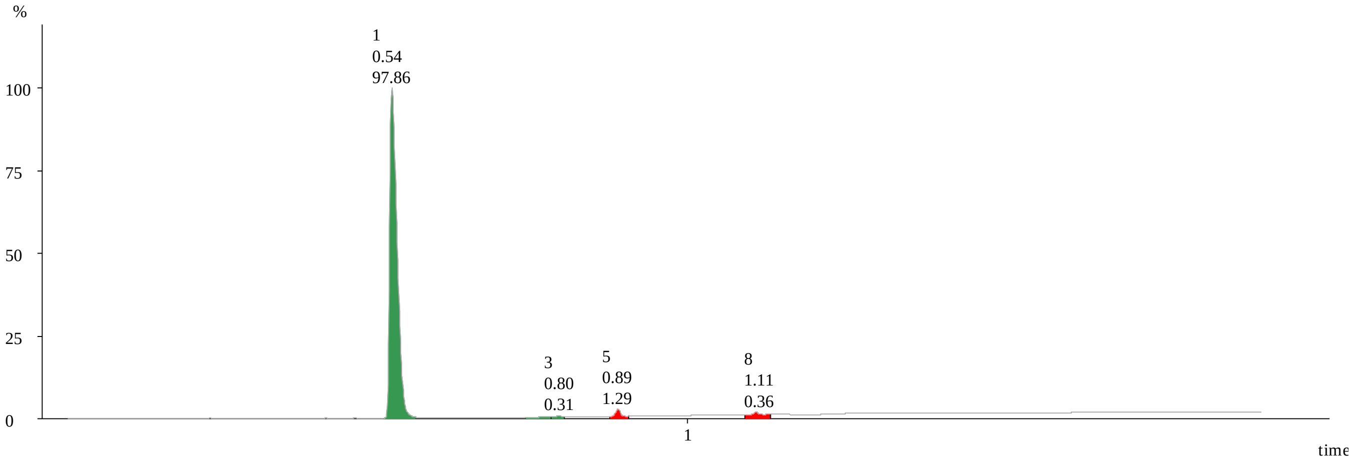

Peak_ID	Peak	Area	Area%	Height	Time	Mass Found
1	0.53 0.58	3.E4	97.86	2.E6	0.54	595.28
2	0.75 0.79	5.E1	0.18	5.E3	0.78	595.28
3	0.79 0.81	8.E1	0.31	1.E4	0.80	595.28
5	0.88 0.91	4.E2	1.29	4.E4	0.89	
8	1.09 1.13	1.E2	0.36	1.E4	1.11	

Sample: 165
File: Ar39292ac_65
Vial: A/9

Date: 21-Jun-2011
Time: 06:03:37
Description: 10009342

AMRI code: ALB-H01006214
Vial label: C1004907AMP0002

MS ES+ :596.28

max. intensity: 3.5E6

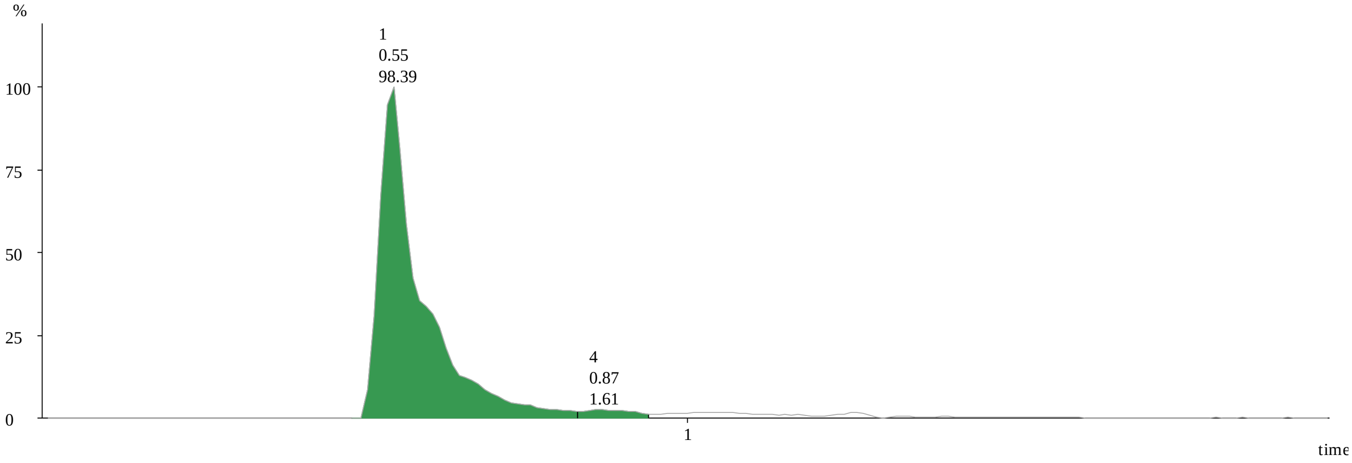

Peak_ID	Peak	Area	Area%	Height	Time	Mass Found
1	0.48 0.83	3.E5	98.39	4.E6	0.55	595.28
4	0.83 0.94	4.E3	1.61	5.E4	0.87	595.28

MS ES+ :TIC

max. intensity: 6.2E6

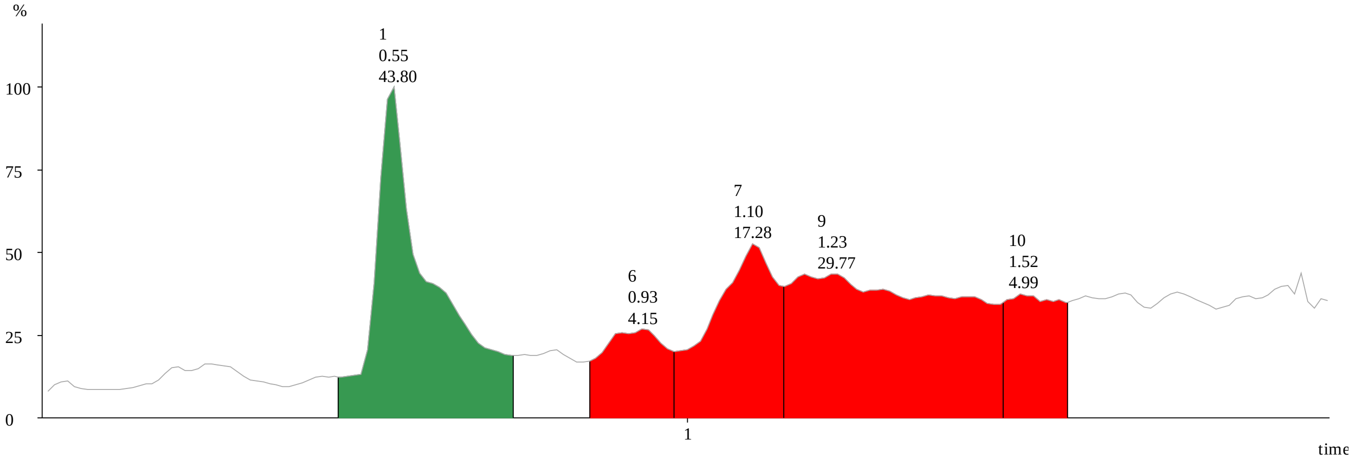

Peak_ID	Peak	Area	Area%	Height	Time	Mass Found
1	0.46 0.73	4.E5	43.8	5.E6	0.55	595.28
6	0.85 0.98	4.E4	4.15	5.E5	0.93	
7	0.98 1.15	2.E5	17.28	2.E6	1.10	
9	1.15 1.49	3.E5	29.77	1.E6	1.23	
10	1.49 1.59	5.E4	4.99	6.E5	1.52	

MS: ES+

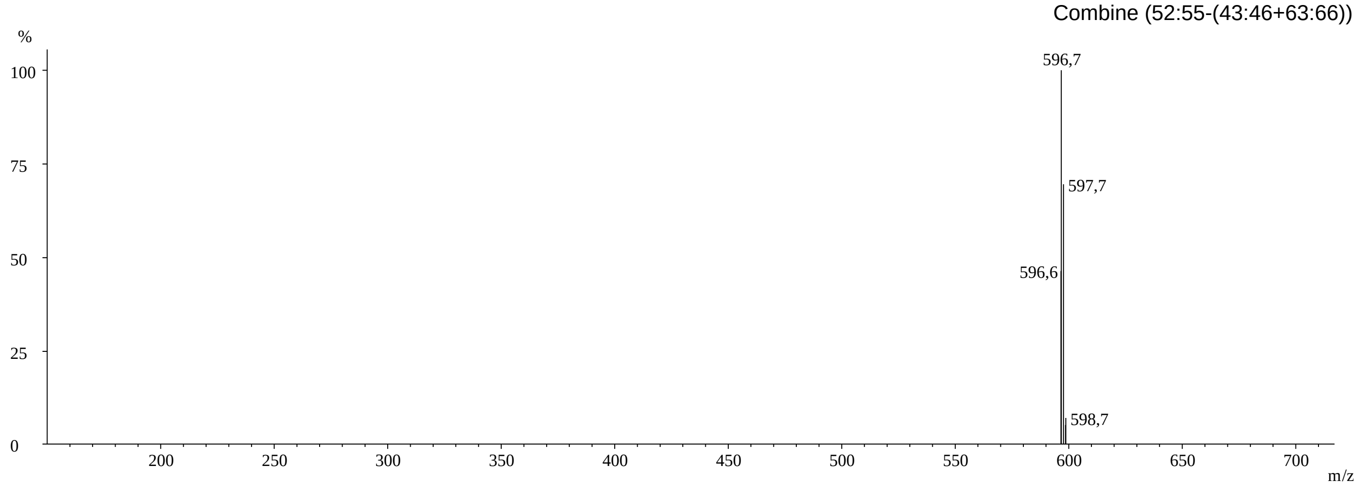

Peak_ID	Compound	Time	Mass found
1	Found	0.54	595.2800

MS: ES+

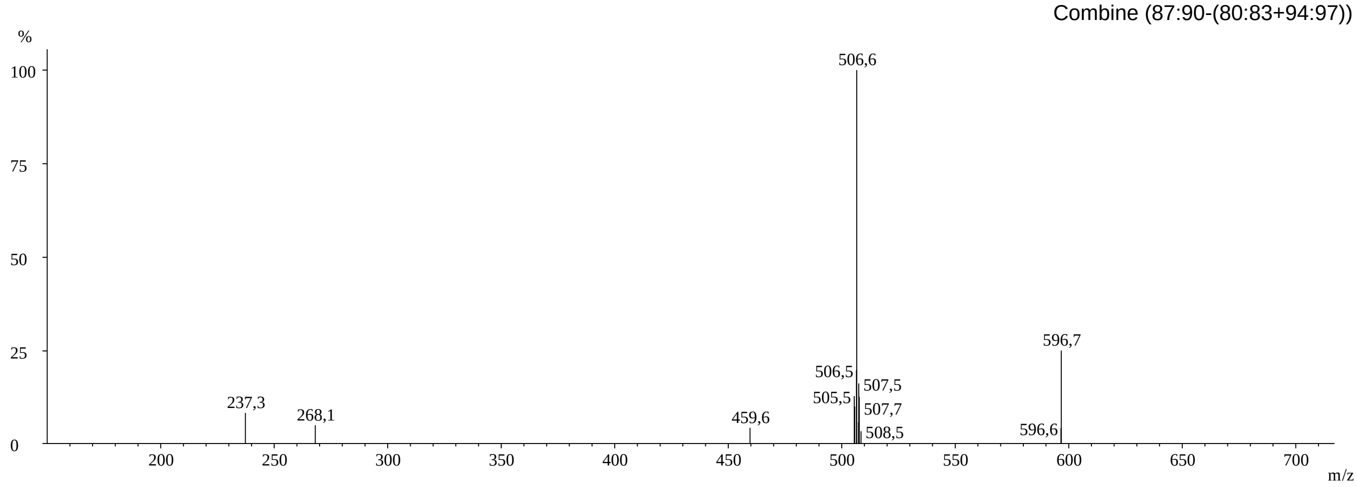

Peak_ID	Compound	Time	Mass found
5		0.89	
