# Supplementary figures and images for: Discovering allatostatin type-C receptor specific agonists
Source: Nat Commun. 2024 May 10;15:3965. doi: 10.1038/s41467-024-48156-w (PMC11087482; doi:10.1038/s41467-024-48156-w)

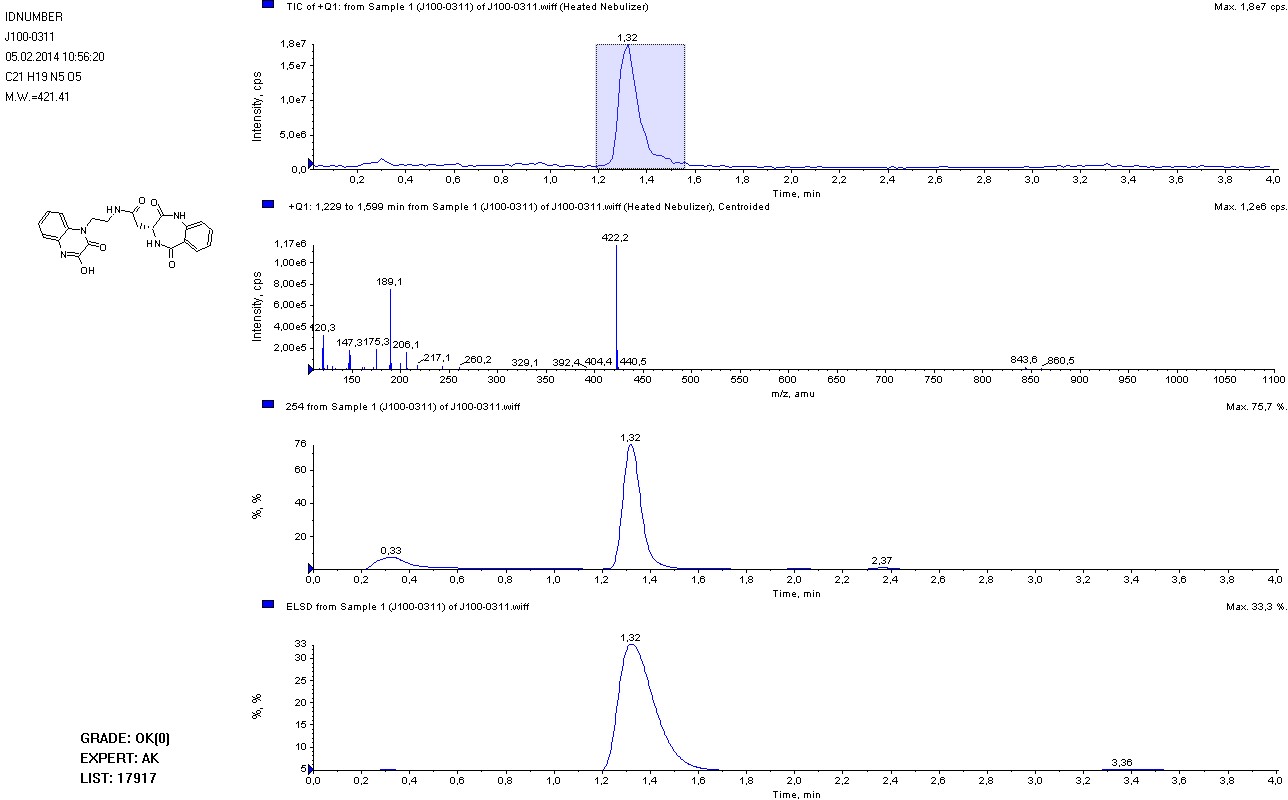

Supplement: Supplementary file 5 — Source Data [file 41467_2024_48156_MOESM5_ESM.zip › source-data/Source_Data/Spectra/FirstSide/J100-0311.JPG]
